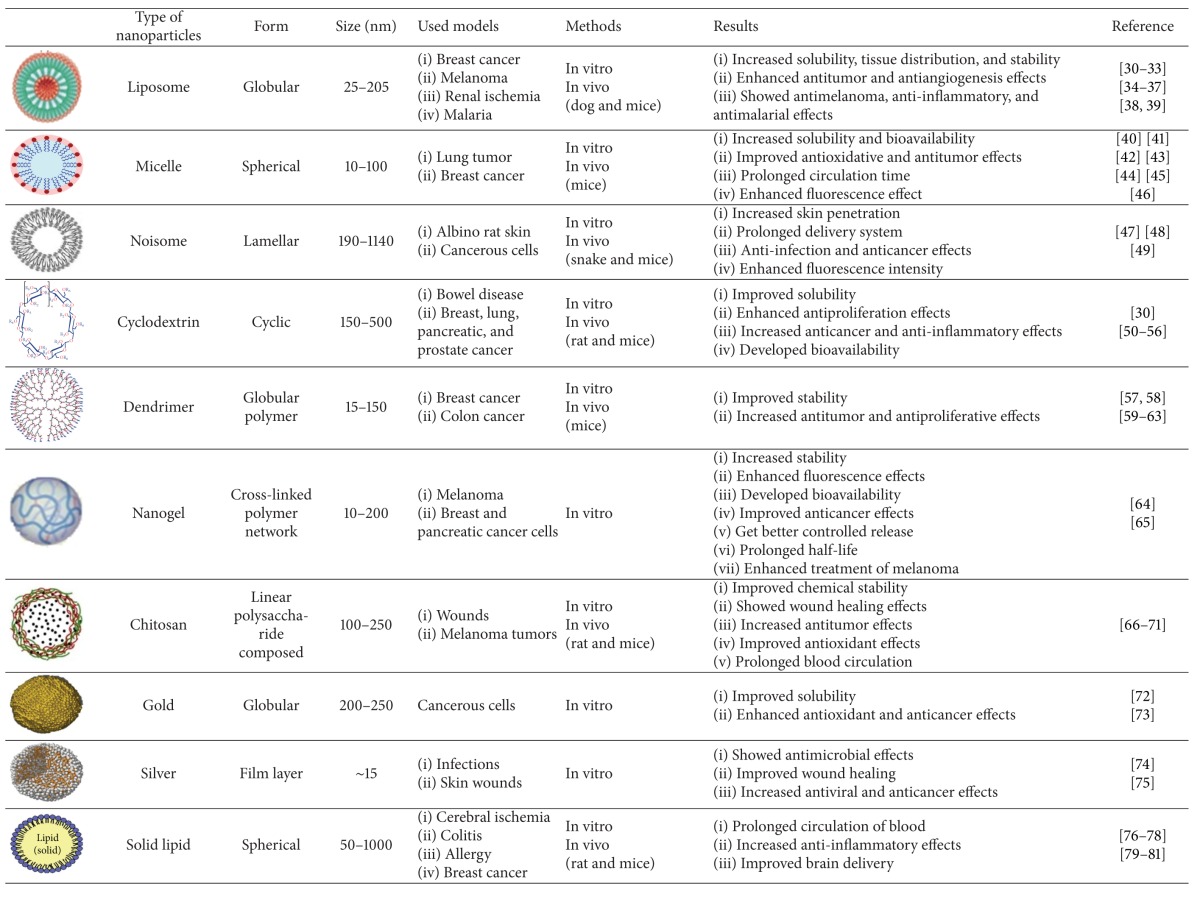# Nanotechnology-Applied Curcumin for Different Diseases Therapy

**DOI:** 10.1155/2014/394264

**Published:** 2014-06-05

**Authors:** Negar Ghalandarlaki, Ali Mohammad Alizadeh, Soheil Ashkani-Esfahani

**Affiliations:** ^1^Cancer Research Center, Tehran University of Medical Sciences, Tehran, Iran; ^2^Student Research Committee, Shiraz University of Medical Sciences, Shiraz, Iran

## Abstract

Curcumin is a lipophilic molecule with an active ingredient in the herbal remedy and dietary spice turmeric. It is used by different folks for treatment of many diseases. Recent studies have discussed poor bioavailability of curcumin because of poor absorption, rapid metabolism, and rapid systemic elimination. Nanotechnology is an emerging field that is potentially changing the way we can treat diseases through drug delivery with curcumin. The recent investigations established several approaches to improve the bioavailability, to increase the plasma concentration, and to enhance the cellular permeability processes of curcumin. Several types of nanoparticles have been found to be suitable for the encapsulation or loading of curcumin to improve its therapeutic effects in different diseases. Nanoparticles such as liposomes, polymeric nanoparticles, micelles, nanogels, niosomes, cyclodextrins, dendrimers, silvers, and solid lipids are emerging as one of the useful alternatives that have been shown to deliver therapeutic concentrations of curcumin. This review shows that curcumin's therapeutic effects may increase to some extent in the presence of nanotechnology. The presented board of evidence focuses on the valuable special effects of curcumin on different diseases and candidates it for future clinical studies in the realm of these diseases.

## 1. Introduction

**Figure 1:**
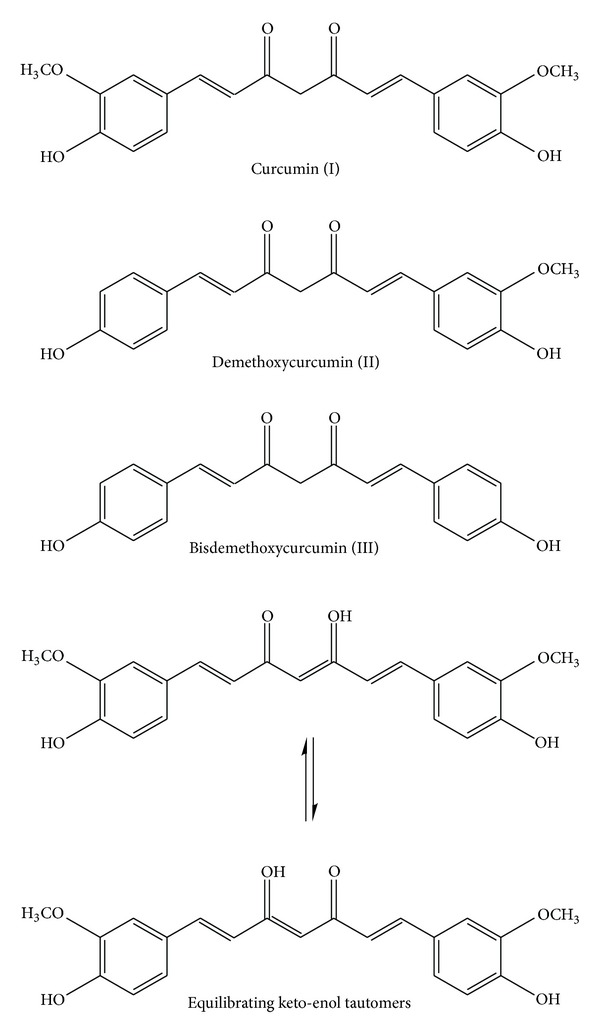
Curcumin I, II, and III (curcumin, demethoxycurcumin, and bisdemethyoxycurcumin) and curcumin keto-enol tautomers.

Curcumin, 1,7-bis(4-hydroxy-3-methoxyphenyl)-1,6-heptadien-3,5-dione, is a lipophilic molecule that rapidly permeates cell membrane [1]. Typical extract of* Curcuma longa* L. contains the structures I to III: (I) diferuloylmethane/curcumin (curcumin I, 75%), (II) demethoxycurcumin (curcumin II, 20%), and (III) bisdemethoxycurcumin (curcumin III, 5%) [2, 3] (Figure 1). Curcumin is an active ingredient in the herbal remedy and dietary spice turmeric [4]. It has a long history of administration by different folks of China, India, and Iran for the treatment of many diseases such as diabetes, liver diseases, rheumatoid diseases, atherosclerosis, infectious diseases, cancers, and digestive disorders such as indigestion, dyspepsia, flatulence, and gastric and duodenal ulcers [5, 6]. Many researchers have worked on curcumin due to its various therapeutic effects on different diseases. Shortly, curcumin has received attention mostly due to its antioxidant, anti-inflammatory, antitumoral, apoptosis-inducing, and antiangiogenesis effects, which were reported in many investigations. It acts on multiple targets in cellular pathways making this agent able to perform multiple actions [7]. The simple molecular structure along with the relative density of functional groups in curcumin provides researchers with an outstanding target for structure-activity relationship and lead optimization studies. The structural analogues of curcumin have been reported to enhance the rate of absorption with a peak plasma half-life [8–10]. Recent investigations have considered curcumin a lead compound for designing new chemotherapeutic agents for treatment of cancers including colon cancers [11], prostate cancers [12], and other conditions with indication of chemotherapy [13, 14].

Curcumin is remarkably well tolerated, but its bioavailability is poor. It does not appear to be toxic to animals [15] or humans [16], even at high doses. Recent studies have discussed poor bioavailability of curcumin because of poor absorption, rapid metabolism, and rapid systemic elimination [17, 18]; however, comprehensive pharmacokinetic data are still missing. In a study done by Yang et al. [19], they reported 1% bioavailability for oral administration of curcumin in rats. On the elimination of curcumin, an investigation in rat model demonstrated that after oral administration of 1 g/kg of curcumin, more than 75% was excreted in feces and negligible amount of curcumin was detected in urine [20]. Additionally, FDA has declared curcumin as “generally safe.” Although curcumin showed a wide variety of useful pharmacological effects and has been found to be quite safe in both animals and humans, there are some studies concerning its toxicity [21]. In spite of these advantages, curcumin has poor water solubility; as a consequence, it reveals solubility-limited bioavailability, which makes it a class II drug in the biopharmaceutics classification system [22]. Additionally, due to its rapid intestinal and hepatic metabolism, about 60% to 70% of an oral dose of curcumin gets eliminated by the feces [23].

As mentioned above, curcumin has been proven to be effective in treatment of different diseases with low toxicity to human and animals. It is extremely safe upon oral administration even at very high doses; however, it is limited due to its poor bioavailability, stability, low solubility, and rapid degradation and metabolism. Overcoming these problems has been the main goal of many studies over the past three decades. Since curcumin was demonstrated to have poor bioavailability and selectivity [17, 24], numerous analogues of this material have been introduced and tested in order to evaluate their activities against known biological targets and to also improve their bioavailability, selectivity, and stability [25–28]. In addition, several approaches were introduced to improve the bioavailability, to increase the plasma concentration, and to enhance the cellular permeability and resistance to metabolic processes of curcumin. Using nanoparticles for targeting drug delivery appeared to provide curcumin with longer circulation, better permeability, and stronger resistance to metabolic processes.

## 2. Nanotechnology Approaches for Curcumin

Nanotechnology is increasingly considered to be the technology of the future. Among the wide applications of nanotechnology is the use of nanoparticles for enhancing the bioavailability and the solubility of lipophilic compounds such as curcumin in drug delivery systems. Therefore, applying nanoparticles gained immense popularity in the last decade due to their potential to improve the therapeutic effects of the encapsulated drugs by protecting drugs from enzymatic degradation, providing their controlled release and prolonged blood circulation, changing their pharmacokinetics, decreasing their toxicity, and limiting their nonspecific uptake [29]. Over a period of time, numerous emphases have been given to develop the biodistribution of natural curcumin, but it is only just recently that the application of the field of nanotechnology has considerably enhanced its therapeutic effects. Nanoparticles such as liposomes, polymeric nanoparticles, micelles, nanogels, niosomes, cyclodextrins, dendrimers, silvers, and solid lipids are emerging as one of the useful alternatives that have been shown to deliver therapeutic concentrations of curcumin. The use of the above nanoparticle has improved main problems of curcumin such as low solubility, instability, poor bioavailability, and rapid metabolism in cancers, wound healing, Alzheimer's disease, epilepticus, ischemia diseases, inflammatory diseases, and so on (Table 1).

## 3. Liposomes

**Figure 2:**
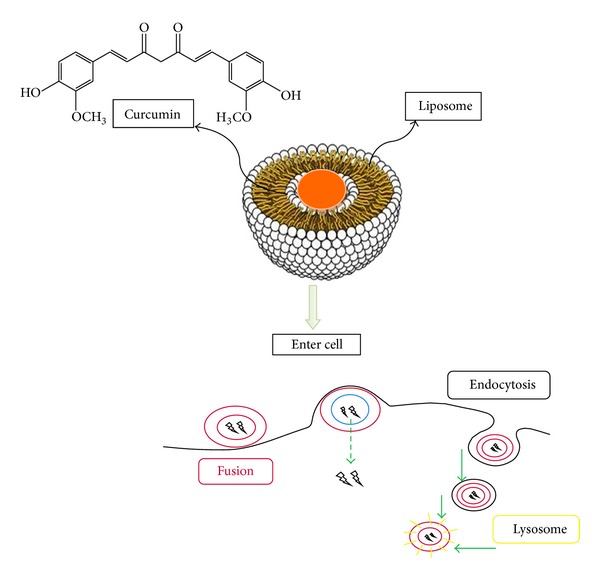
A schematic figure of how curcumin is located in liposomes and enters into cells. Curcumin is encapsulated inside the liposomal container and covalently bound to liposome, so it is protected from destruction on the way to the target. The liposome membrane is usually made of phospholipids, which constitute biological membranes and can deliver curcumin into cells by two different ways: fusion and endocytosis.

Liposomes are synthetic vesicles with globular character that can be produced from natural phospholipids [82]. They are self-assembling closed colloidal constructions composed of lipid bilayers, and they have a spherical shape in which an outer lipid bilayer surrounds a central aqueous space [83]. The liposome diameter varies from 25 nm to 2.5 mm (Table 1). They are stated to act as immunological adjuvants and drug carriers. Liposomes can encapsulate drugs with widely varying solubility or lipophilicity, entrapped either in the aqueous core of the phospholipid bilayer or at the bilayer interface [84]. Moreover, they are able to deliver drugs into cells by fusion or endocytosis, and practically any drug, irrespective of its solubility, can be entrapped into liposomes (Figure 2). In this regard, to enhance the solubility of curcumin, Rahman et al. [30] prepared *β*-cyclodextrin-curcumin inclusion complexes that entrapped both native curcumin and the complexes separately into liposomes. All curcumin-containing formulations were effective in inhibiting cell proliferation in in vitro cell culture. In another study, Shi et al. [31] developed a water-soluble liposomal curcumin to examine curcumin's preventive effects on lung fibrosis via intravenous administration in mice by using enzyme-linked immunosorbent assay method (ELISA). Results showed that liposomal curcumin can effectively diminish radiation pneumonitis and fibrosis of lung and sensitize LL/2 cells to irradiation. These data suggest that the systemic administration of liposomal curcumin with enhanced solubility is safe and deserves to be investigated for further clinical application.

Some studies showed that the drugs encapsulated in liposomes are expected to be transported without rapid degradation and result in minimum side effects and show more signs of stability in the recipients. In this regard, to assess curcumin tissue distribution, Matabudul et al. [32] questioned whether different durations of intravenous infusions of Lipocurc can alter curcumin metabolism and its tissue distribution and whether treating necropsied tissues of Beagle dogs with phosphoric acid prior to measuring curcumin and its metabolite (tetrahydrocurcumin) can stabilize the compounds allowing for accurate analytical measurements. Results demonstrated that the addition of liposomes may inhibit or saturate a putative reductase enzyme that converts curcumin to tetrahydrocurcumin and stabilizes the levels of curcumin. Tetrahydrocurcumin in some tissues (lung, spleen, and liver), but not all the examined tissues (lung, spleen, liver, pancreas, kidney, and urinary bladder), raised issues of tissue-specific curcumin and tetrahydrocurcumin stability via a transporter-dependent mechanism that elevated tissue concentrations of curcumin. Additionally, to obtain better understanding of curcumin interaction mechanisms with lipid membranes and improve the stability of curcumin, Karewicz et al. [33] banded curcumin to egg yolk phosphatidylcholine, dihexadecyl phosphate and cholesterol, then in order to determine curcumin binding constant to liposomes they used absorption and fluorescence techniques. The egg yolk phosphatidylcholine/dihexadecyl phosphate/cholesterol liposomal bilayer curcumin stabilized the system proportionally to its content, while the egg yolk phosphatidylcholine/dihexadecyl phosphate system destabilized upon drug loading. The three-component lipid composition of the liposome seems to be the most promising system for curcumin delivery. Furthermore, an interaction of free and liposomal curcumin with egg yolk phosphatidylcholine and mixed monolayers was also studied by using Langmuir balance measurements. Condensing effects of curcumin on egg yolk phosphatidylcholine and egg yolk phosphatidylcholine/dihexadecyl phosphate monolayers and loosening influence on egg yolk phosphatidylcholine/dihexadecyl phosphate/cholesterol ones were observed. It was also demonstrated that curcumin-loaded egg yolk phosphatidylcholine liposomes are more stable upon interaction with the model lipid membrane than the unloaded ones. In another study, Chen et al. [85] reported the effects of different liposomal formulations on curcumin stability in phosphate buffered saline, human blood, plasma, and culture medium. Liposomal curcumin showed a higher stability than free curcumin in phosphate buffered saline (PBS). Liposomal and free curcumin showed similar stability in human blood plasma and culture medium. In addition, results on the toxicity of concanavalin-A showed that dimyristoylphosphatidylcholine and dimyristoylphosphatidylglycerol were toxic on lymphoblastoid cell lines. However, addition of cholesterol to the lipids at dimyristoylphosphatidylcholine/dimyristoylphosphatidylglycerol/cholesterol almost completely eliminated the lipid toxicity to these cells. Liposomal curcumin had similar or even stronger inhibitory effects on concanavalin-A-stimulated human lymphocyte, splenocyte, and lymphoblastoid cell proliferation. They concluded that liposomal curcumin may be useful for intravenous administration to improve the bioavailability and efficacy, facilitating the in vivo studies that could ultimately lead to clinical application of curcumin.

In addition, liposomal curcumin's potential was evaluated against cancer models of osteosarcoma and breast cancer by Dhule et al. [34] with curcumin-loaded *γ*-cyclodextrin liposomal nanoparticles. The results showed promising anticancer potential of liposomal curcumin both in vitro and in vivo against osteosarcoma and breast cancer cell lines via the caspase cascade that leads to apoptotic cell death. The efficiency of the liposomal curcumin nanoparticles was also confirmed by using a xenograft osteosarcoma model in vivo. Li et al. [9] encapsulated curcumin in a liposomal delivery system for intravenous administration. They also showed the liposome-encapsulated curcumin effects on proliferation, apoptosis, signaling, and angiogenesis by using human pancreatic carcinoma cells in vitro and in vivo. Liposome-encapsulated curcumin suppressed pancreatic carcinoma growth in murine xenograft models and inhibited tumor angiogenesis in vivo. It also downregulated the NF-*κ*B pathway, suppressed growth, and induced apoptosis of human pancreatic cells in vitro and showed antitumor and antiangiogenesis effects in vivo [35, 36]. Chen et al. [37] studied in vitro skin permeation and in vivo antineoplastic effects of curcumin by using liposomes as the transdermal drug-delivery system. Curcumin-loaded liposomes exhibited ability to inhibit the growth of melanoma cells. A considerable effect on antimelanoma action was detected with curcumin-loaded liposomes. These results, similar to the results of other studies, suggest that liposomes would be a hopeful delivery service for curcumin in cancer management [30, 86, 87]. These data indicate a significant liposomal curcumin potential as delivery vehicles for the treatment of different cancers (Table 1).

Rogers et al. [38] also administered liposomes containing curcumin to target delivery to renal tubular epithelial and antigen-presenting cells in mice renal ischemia model. Liposomal curcumin significantly improved serum creatinine, reduced histological injury and cellular apoptosis, and lowered toll-like receptor-4, heat shock protein-70, and tumor necrosis factor alpha (TNF-*α*) mRNA expression, and it also decreased neutrophil infiltration and inflammatory interleukins expression. In this regard, Basnet et al. [39] developed vaginal administration of liposomal curcumin. Liposomal curcumin was found to be twofold to sixfold more potent than corresponding free curcumin. Results showed that liposomal delivery systems enhance anti-inflammatory properties of curcumin. Also, evaluation of liposomal curcumin cytochrome P450 inhibition was conducted by Mach et al. [88] in liver tissues. Results demonstrated that there is low potential for CYP450 mediated drug interactions at physiologic serum concentrations of liposomal curcumin. It will not interact with other chemotherapy agents that are metabolized and/or eliminated via the primary drug metabolizing cytochrome P450 pathways [88].

The therapeutic efficacies of novel liposomal delivery systems based on artemisinin or artemisinin-based combination therapy with curcumin have been investigated and reported by Isacchi et al. [89]. They reported that artemisinin alone began to decrease parasitaemia levels only 7 days after the start of the treatment, and it appears to have a fluctuant trend in blood concentration which is reflected in the antimalarial effectiveness. By contrast, treatments with artemisinin loaded with liposomal delivery systems appeared to have an immediate antimalarial effect which cured all malaria-infected mice within the same postinoculation period of time. In particular, artemisinin loaded with liposomal curcumin seems to give the most pronounced and statistically significant therapeutic effect in this murine model of malaria. The enhanced permanency in blood of artemisinin loaded with liposomal curcumin suggests application of these nanosystems as suitable passive targeted carriers for parasitic infections [89]. This strong effect of formulation is added up to the mechanism of action of artemisinin which acts in the erythrocyte cycle stage of human host as a blood schizonticide. Agarwal et al. [90] also assessed the acute effects of liposome-entrapped curcumin on increasing current electroshock seizures, pentylenetetrazole-induced seizures, and status epilepticus in mice. Liposome-entrapped curcumin demonstrated significant increase in seizure threshold current and latency to myoclonic and generalized seizures increasing current electroshock and pentylenetetrazole-induced seizures, respectively. It also increased the latency to the onset and decreased the duration of seizures during status epilepticus. Therefore, liposomal-entrapped curcumin can possess anticonvulsant activity against status epilepticus in mice (Table 1).

To put it briefly, the above data suggest that the administration of liposomal curcumin has numerous beneficial effects which could lead to required clinical applications. These better outcomes take place by means of enhanced solubility, more safety and minimum side effects, more signs of stability in the blood, increased bioavailability and efficacy, owning a potential role as delivery vehicles for the treatment of different cancers, potent anti-inflammatory and antimalaria response, and, finally, anticonvulsant activity.

## 4. Micelles

A typical micelle is a surfactant molecule aggregate dispersed in a liquid colloid. It is a nanosized vesicular membrane which becomes soluble in water by gathering the hydrophilic heads outside in contact with the solvent and hydrophobic tails inside, which is known as emulsification. Micelles are lipid molecules that arrange themselves in a spherical form in aqueous solutions with a very narrow range from 10 to 100 nm in size, which makes them more stable toward dilution in biological fluids [84]. The shape or morphology of micelles is from amphiphilic block copolymers such as spherical, rodlike, and starlike, as well as vesicles (Table 1). The self-assembly of amphiphilic block copolymer is a reversible process, and the shape varies with the copolymers' composition and length ratio [91]. The functional properties of micelles are based on amphiphilic block copolymers, which come together to form a nanosized core/shell structure in aqueous media. The hydrophobic core area hands out as a pool for hydrophobic drugs, while the hydrophilic shell area stabilizes the hydrophobic core and makes the polymers water soluble. Polymeric micelles can serve as transporters of water-insoluble drugs such as curcumin, which can augment the drug's efficiency by targeting definite cells or organs; therefore, fewer drugs accumulate in healthy tissues and their toxicity reduces, and occasionally higher doses can be administered [92]. In this regard, to overcome the poor water solubility of curcumin, Liu et al. [93] prepared curcumin-loaded biodegradable self-assembled polymeric micelles by solid dispersion method, which was simple and easy to scale up. Release profile showed a significant difference between rapid release of free curcumin and much slower and sustained release of curcumin-loaded micelles. In addition, the preparation of curcumin-loaded micelles based on amphiphilic Pluronic/polycaprolactone block copolymer was investigated by Raveendran et al. [40], which proved to be efficient in enhancing curcumin's aqueous solubility. Some other studies also deliberated on highly surface-active compounds such as poloxamers or Pluronic that can self-assemble into spherical micelle. In vitro results showed that spherical curcumin-loaded mixed micelles might serve as a potential nanocarrier to improve the solubility and biological activity of curcumin [94–96]. In another study, the aqueous solubility of the curcumin was increased by encapsulation within the micelles [97]. Solubilization was directly related to the compatibility between the solubilizate and polycaprolactone as determined by the Flory-Huggins interaction parameter. Molecular modeling study suggested that curcumin tended to interact with polycaprolactone serving as a core embraced by polyethylene glycol as a shell. In addition, Yu et al. [41] showed the structure of modified *ε*-polylysine micelles and their application in improving cellular antioxidant activity of curcuminoids. Results of their investigation revealed that modified *ε*-polylysine micelles were able to encapsulate curcuminoids and improve their water solubility and cellular antioxidative activity compared with free curcuminoids. They suggested that these micelles may be used as new biopolymer micelles for delivering poorly soluble drugs such as curcumin. Another study synthesized curcumin in sodium dodecyl sulfate and cetyltrimethylammonium bromide micelles to overcome the poor water solubility of curcumin and demonstrated antioxidative effects of curcumin analogues against the free-radical-induced peroxidation of linoleic acid in these micelles [98, 99]. Kinetic analysis of the antioxidation processes demonstrated that these compounds exhibited extraordinarily higher antioxidative activity in micelles due to their solubility being higher than free curcumin [98].

Drug release from micelles is governed by different issues including micelle stability, the rate of copolymer biodegradation, and drug diffusion. By the way, Sahu et al. [100] reported the potential of the two most common Pluronic triblock copolymer micelles, Pluronic F127 and F68, for curcumin encapsulation efficiency and stability. Pluronic F127 showed better encapsulation efficiency and good stability for long-term storage than Pluronic F68. Atomic force microscopy (AFM) study revealed that the drug-encapsulated micelles are spherical in shape with diameters below 100 nm. Pluronic-encapsulated curcumin demonstrated slower and sustained release of curcumin from the micelles and considerable anticancer activity in comparison with free curcumin in vitro cytotoxicity study. In addition, Podaralla et al. [42] reported a natural protein core-based polymeric micelle and demonstrated its application for the delivery of hydrophobic anticancer drugs, specifically curcumin. They synthesized novel biodegradable micelles by conjugating methoxy polyethylene glycol and zein, a biodegradable hydrophobic plant protein which can be found in Maize, and then encapsulating with curcumin. Polyethylene glycol zein micelles sustained the curcumin release up to 24 hrs in vitro and significantly enhanced its aqueous solubility and stability with the 3-fold reduction in IC50 value of curcumin. So, since the curcumin is finely protected from possible inactivation by their micellar surroundings, its retention and bioavailability can be enhanced (Table 1).

Aiming to modify the pharmacokinetics of curcumin, Song et al. [43] synthesized a poly(D,L-lactide-co-glycolide)-b-poly(ethylene glycol)-b-poly(D,L-lactide-co-glycolide) (PLGA-PEG-PLGA) with micelles. PLGA-PEG-PLGA micelles provided higher area under the concentration curve (AUC) and enhanced residence time, clearance, and distribution half-life in comparison with curcumin solution. The prolongation of half-life, enhanced residence time, and decreased total clearance indicated that curcumin-loaded micelles could prolong acting time of curcumin in vivo. These results may be related to the curcumin location within the micelles and increased viscosity of copolymer solution at the body temperature. The variation of AUC indicated that the curcumin-loaded micelles provided higher bioavailability than curcumin solution, and the biodistribution study showed that the micelles had decreased drug uptake by liver and spleen and enhanced drug distribution in lung and brain. These results suggested that PLGA-PEG-PLGA micelles would be a potential carrier for curcumin. In addition, Ma et al. [94] demonstrated the pharmacokinetics of both solubilized curcumin and its polymeric micellar formulation in rats by using a simple, rapid, and reliable HPLC method. They concluded that encapsulation of curcumin in the polymeric micellar formulation led to increase in curcumin's half-life and distribution volume.

In addition, curcumin-micelles can be affected by physicochemical characteristics, concentration, and location within the micelles. The polymeric micelles have a prolonged circulation time due to their small size and hydrophilic shell that reduce the drug uptake by the mononuclear phagocyte system [101]. Leung et al. [44] reported that encapsulated curcumin in cationic micelles suppresses alkaline hydrolysis that was studied in three types of micelles composed of the cationic surfactants cetyltrimethylammonium bromide (CTAB) and dodecyltrimethylammonium bromide (DTAB) and the anionic surfactant sodium dodecyl sulfate (SDS). Curcumin underwent rapid degradation in the SDS micellar solution by alkaline hydrolysis at pH of 13, while it was significantly suppressed with a yield of suppression close to 90% in the presence of either CTAB or DTAB micelles. Results from fluorescence spectroscopic studies revealed that curcumin is dissociated from the SDS micelles to the aqueous phase at this pH while curcumin remains encapsulated in CTAB and DTAB micelles at pH 13. The absence of encapsulation and stabilization in the SDS micellar solution resulted in rapid hydrolysis of curcumin. Some other studies showed other curcumin-loaded micelles properties. Wang et al. [102] introduced the sensitive fluorometric method for the determination of curcumin using the enhancement of mixed micelle. This method had the advantages of high sensitivity, selectivity, and stability. The fluorescence of curcumin was greatly enhanced by mixed micelle of sodium dodecylbenzenesulfonate and cetyltrimethylammonium bromide (SDBS-CTAB). This study indicated that fluorescence quantum yield of curcumin in SDBS-CTAB micelle was about 55-fold larger than that of aqueous solution containing 1.0% ethanol, which was in agreement with their fluorescence intensity ratio. As a result, curcumin can be used as a fluorophore in fluorescence polarization anisotropy measurement to determine the critical micellar concentration of surfactant and to study the interaction between them. In addition, Adhikary et al. [45] performed femtosecond fluorescence upconversion experiments on the naturally occurring medicinal pigment, curcumin, in anionic, cationic, and neutral micelles. These micelles were composed of SDS, dodecyltrimethylammonium bromide (DTAB), and Triton X-100. They revealed the curcumin's excited-state kinetics in micelles with fast (3–8 ps) and slow (50–80 ps) components. While deuteration of curcumin had a negligible effect on the fast component, the slow component exhibited a pronounced isotope impact of approximately 1.6, which indicates that micelle-captured curcumin undergoes excited-state intramolecular hydrogen atom transfer. Moreover, Began et al. [46] had attached curcumin to phosphatidylcholine micelles followed by fluorescence measurements. Curcumin in aqueous solution did not inhibit dioxygenation of fatty acids by lipoxygenase 1, but it inhibited the oxidation of fatty acids when bound to phosphatidylcholine micelles. Results demonstrated that 8.6 *μ*M of curcumin bound to the phosphatidylcholine micelles is required for 50% inhibition of linoleic acid peroxidation. Lineweaver-Burk plot analysis had indicated that curcumin is a competitive inhibitor of lipoxygenase 1 with Ki of 1.7 *μ*M for linoleic acid and 4.3 *μ*M for arachidonic acid, respectively. By using spectroscopic measurement, they revealed that the inhibition of lipoxygenase 1 activity by curcumin can be due to binding to active center iron and curcumin after binding to the phosphatidylcholine micelles acts as an inhibitor of lipoxygenase 1. In a recent investigation, the critical micelle concentration of the amphiphilic polymer was determined by using fluorescent probe. Outcomes indicated that Pluronic/polycaprolactone micelles may be a promising candidate for curcumin delivery to cancer cells of colorectal adenocarcinoma [40]. In another pharmacokinetic study, curcumin micelles demonstrated higher concentration and longer retention time in plasma and tumor sites, so they had stronger inhibitory effects on proliferation, migration, invasion, and tube formation of carcinoma cells than free curcumin; for example, curcumin micelles were shown to be more effective, presumably due to higher concentration in inhibiting tumor growth and prolonged survival in both subcutaneous and pulmonary metastatic tumor models [103].

Investigating the influence of micelles on cytotoxicity of curcumin, specifically in cancer therapy, in vitro study by Raveendran et al. [40] showed that Pluronic/polycaprolactone micelles could be a promising candidate for curcumin delivery to cancer cells regarding the cytotoxicity and cellular uptake of the curcumin-loaded micelles in colorectal adenocarcinoma cells. An investigation by Wang et al. [104] revealed that the encapsulated curcumin maintains its potent antitumor effects; however, curcumin-loaded micelles were more effective in inhibiting tumor growth and spontaneous pulmonary metastasis in subcutaneous 4T1 breast tumor model and prolonged survival of tumor-bearing mice. Immunofluorescent and immunohistochemical studies also showed that tumors of curcumin-loaded micelle-treated mice had more apoptotic cells, fewer microvessels, and fewer proliferation-positive cells [104]. In addition, Yang et al. [19] had conjugated methoxypolyethylene glycol-polylactic acid (mPEG-PLA) micelle to multiple curcumin molecules; the cytotoxicity study results showed that the effect of IC50 of mPEG-PLA-Tris-curcumin on human hepatocellular carcinoma cells was similar to unmodified curcumin. The cellular uptake study demonstrated that these carriers could successfully transport the drug to the cytoplasm of hepatic cells. Micelles containing multiple drug molecules were an efficient means to increase loading and intracellular delivery of low-potency curcumin [19]. Moreover, Mohanty et al. [105] reported that curcumin encapsulated in methoxy poly(ethylene glycol)/poly-epsilon-caprolactone diblock copolymeric (MePEG/PCL) micelle, by varying the copolymer ratio (40 : 60 MePEG/PCL ratio was selected due to its high encapsulation), had increased bioavailability due to intensified uptake, 2.95 times more, with comparative cytotoxic effects by induction of apoptosis in contrast with unmodified curcumin at equimolar concentrations. Overall, these data obviously showed the commitment of a micellar system for efficient solubilization, stabilization, and controlled delivery of the hydrophobic drug, such as curcumin, for cancer therapy.

Concisely, curcumin-loaded micelles can boost the drug's efficiency by targeting definite cells and result in less drug accumulation in healthy tissues and reduction of toxicity. Curcumin's aqueous solubility and much slower and sustained release of drug caused by curcumin-loaded micelles also get in use in several conditions. The retention and bioavailability of curcumin could be elevated since the curcumin is protected from possible inactivation by its micellar surroundings. Locating the curcumin in the micelles can also enhance half-life and residence time and decrease total clearance leading to prolongation of acting time of curcumin. Curcumin micelles can be influenced by physicochemical features including their size and electrical charges, concentration, and location within the micelles. These data obviously showed the commitment of a micellar system for efficient solubilization, stabilization, and controlled delivery of the hydrophobic drug, such as curcumin, for cancer therapy (Table 1).

## 5. Niosomes

Niosomes are microscopic lamellar constructions of nonionic surfactant of alkyl or dialkyl polyglycerol ether category with cholesterol that were first introduced in the 70s [106, 107]. Niosomes can provide a container for drug molecules with a wide range of solubilities due to presence of hydrophilic, amphiphilic, and lipophilic moieties in the constitution (Table 1). They behave similar to liposomes in vivo and can be used as an effective alternative to liposomal drug carriers, and those properties depend on the composition of the bilayer as well as the method of their production [108]. Surfactant type, encapsulated drug nature, storage temperature, detergents, and use of membrane spanning lipids can affect niosomes stability [107]. Niosomes are also planned for use in a number of potential therapeutic applications, such as anticancer and anti-infective drug targeting agents [84]. They can improve the therapeutic indices of drugs by restricting their action on the target cells. They also improve oral bioavailability of poorly absorbed drugs such as curcumin to design the novel drug delivery system and increase the skin penetration of drugs [47]. In this regard, in an in vitro study which was performed using albino rat skin, proniosomes of curcumin were prepared by encapsulation of the drug in a mixture of Span 80, cholesterol, and diethyl ether to investigate transdermal drug delivery system [109]. The planned systems distinguished between size, drug entrapment, repose angle, hydration rate, and vesicular stability under different storage settings. Results showed that proniosomes are very stable and promising prolonged delivery systems for curcumin [109]. Mandal et al. [48] also designed a comparative study with different microenvironments for photophysical properties of curcumin inside niosomes by means of steady state, time resolved fluorescence spectroscopy, and dynamic light scattering techniques. Outcomes showed that more rigid and confined microenvironments of niosomes improve the steady state fluorescence intensity along with the fluorescence lifetime of curcumin. The data indicated that niosomes are a good tool for delivery system to suppress the level of degradation of curcumin [48]. In another study, by Rungphanichkul et al., curcuminoid niosomes were developed with a series of nonionic surfactants to enhance skin permeation of curcuminoids. [49]. Results were evaluated based on entrapment efficiency and in vitro penetration of curcuminoids via snake skin. Niosomes drastically enhanced permeation of curcuminoids compared with a vehicle solution of curcuminoids [49]. The fluxes of curcumin, desmethoxycurcumin, and bisdesmethoxycurcumin also were consistent with the qualified hydrophobicity of curcumin, desmethoxycurcumin, and bisdesmethoxycurcumin, respectively. Data indicated that curcuminoids can be fruitfully prepared as niosomes, and such formulations have superior properties for transdermal drug delivery system [49].

Briefly, niosomes can be a potential delivery system for curcumin in order to suppress the degradation of this agent and increase its life time. It has also been demonstrated that niosomes boost the permeation of curcumin through skin (Table 1).

## 6. Cyclodextrins

Cyclodextrins (Cds) are a family of complexes prepared from sugar molecules bound together in cyclic oligosaccharides [110]. They are created from starch by using enzymatic switch. Cds are cyclic oligomers of glucose that can form water-soluble inclusion complexes with small molecules and portions of large complexes [111]. They are exceptional molecules with pseudoamphiphilic construction, which are used industrially in pharmaceutical requirements [84]. Cds are also used in agriculture and in environmental engineering in food, drug delivery systems, and chemical industries [110]. They have an interior hydrophobic surface which can provide a place for residence of poorly water-soluble molecules, while the external hydrophilic area makes its solubility possible in the aqueous setting with high stability (Table 1).

**Figure 3:**
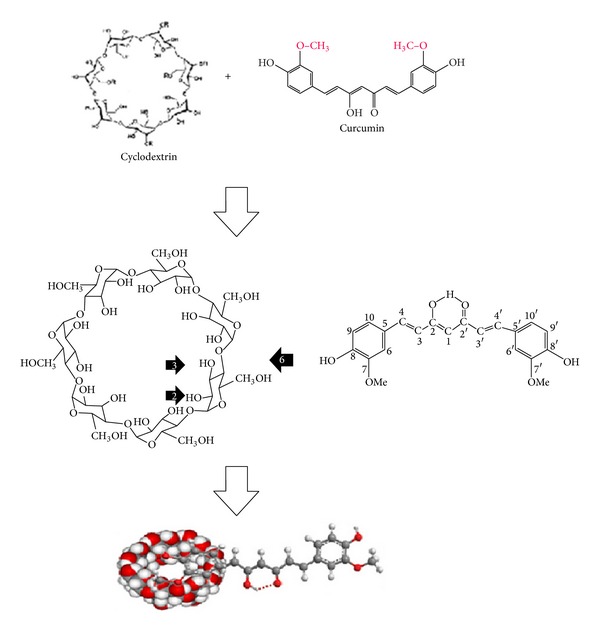
A schematic figure of curcumin connection to the cyclodextrin nanoparticles.

To improve the water solubility and the hydrolytic stability of curcumin, Tønnesen et al. [50] prepared cyclodextrin-curcumin complexes by using HPLC and UV/VIS scanning spectrophotometer techniques [50] (Figure 3). Results showed that the hydrolytic stability of curcumin was sturdily improved by the complex, and also the photodecomposition rate was enhanced in organic solvents compared to the free curcumin. As a result, the cavity size and charge of cyclodextrin side-chains influenced the stability and degradation rate of curcumin [50]. In addition, other investigations on the solubility, phase distribution, and hydrolytic and photochemical stability of curcumin showed that curcumin derivatives were more stable towards hydrolytic degradation in cyclodextrin solutions than free curcumin [51]. The photochemical studies illustrated that curcumin is universally more stable than its other derivatives. Solubility and phase-distribution studies showed that curcuminoids with side groups on the phenyl moiety have higher affinity for the hydroxypropyl-*γ*-cyclodextrin (HP-*γ*-CD) than the cyclodextrins. The radical scavenging investigations confirmed that curcumin is more active than its curcuminoids derivatives, and the free phenolic hydroxyl group may possibly be necessary for the scavenging properties [51]. In another study, to increase the solubility of curcumin, Darandale and Vavia [52] employed cyclodextrin-based nanosponges; they formulated the complex of curcumin with *β*-cyclodextrin nanosponge obtained with dimethyl carbonate as a cross-linker. The loaded nanosponges have shown more solubilization efficiency compared to free curcumin and *β*-cyclodextrin complex. The characterization of curcumin nanosponge complex confirmed the interactions of curcumin with nanosponges. Moreover, in vitro drug release of curcumin was controlled over a prolonged time period, and the complex was nonhemolytic [52]. Therefore, it seems that CDs are permitting vehicles that can be used for oral delivery to develop the bioavailability of insoluble drugs by molecular dispersion and degradation protection and for intravenous delivery to supply as solubilizers for multifaceted hydrophobic drugs without altering their pharmacokinetic properties [84].

Yadav et al. [53] developed a new cyclodextrin complex of curcumin to increase solubility of curcumin and studied its anti-inflammatory and antiproliferative effects. They showed that cyclodextrin-curcumin complex was more active than free curcumin in inhibiting the inflammatory transcription factor, such as nuclear factor kappa-b (NF-*κ*B). In addition, it suppressed cyclin D1 as a cell proliferation marker, matrix metallopeptidase 9 (MMP-9) as an invasion marker in metastasis, and vascular endothelial growth factor (VEGF) as an angiogenesis marker. Cyclodextrin-curcumin complex was also more active in inducing the death receptors and apoptosis of leukemic cells as well as other cancer cell lines. These suggest that cyclodextrin-curcumin complex has superior characteristics compared to free curcumin for cell uptake and antiproliferative and anti-inflammatory effects [53]. Yadav et al. [54] have also planned curcumin complexes by common methods to evaluate the anti-inflammatory effects of cyclodextrin-curcumin complex for the treatment of inflammatory bowel disease (IBD) in an animal rat model. In vivo results showed that curcumin has higher affinity for hydroxypropyl-*β*-cyclodextrin than other cyclodextrins. In addition, hydroxypropyl-*β*-cyclodextrin-curcumin complex proved to be a powerful antiangiogenesis complex. In vivo data also confirmed that the scale of colitis was appreciably attenuated by cyclodextrin-curcumin. In summary, cyclodextrin complex was shown to be valuable in the therapeutic approaches for IBD patients being a nontoxic natural dietary yield [54].

Additionally, Cds can augment bioavailability of insoluble drugs such as curcumin by rising drug solubility and dissolution [84]. They also amplify the permeability of hydrophobic agents by making them accessible at the surface of the membrane's biological barrier. A *β*-cyclodextrin-encapsulated curcumin drug delivery system was developed by Yallapu and colleagues in order to get better curcumin hydrophilic and drug delivery characteristics [55]. Encapsulated-curcumin efficiency was shown to be improved through increasing the ratio of curcumin to cyclodextrin. Then, an optimized cyclodextrin-curcumin complex was assessed for intracellular uptake and anticancer effects. Cell proliferation and clonogenic examinations showed that *β*-cyclodextrin-curcumin self-assembly augmented curcumin delivery and improved its therapeutic efficacy in prostate cancer cells [55]. Moreover, curcumin-loaded *γ*-cyclodextrin liposomal nanoparticles as delivery vehicles were also explored by Dhule et al. [34] and evaluated against cancer models. The resulting 2-hydroxypropyl-*γ*-cyclodextrin/curcumin-liposome complex showed promising anticancer potential both in vitro and in vivo against osteosarcoma and breast cancer. Liposomal curcumin initiated the caspase cascade that led to apoptotic cell death in vitro. In addition, the efficiency of the liposomal curcumin formulation was confirmed in vivo by using a xenograft osteosarcoma model. Data showed that curcumin-loaded *γ*-cyclodextrin liposomes indicated considerable potential as delivery vehicles for cancer cure [34]. Rahman et al. [30] prepared *β*-cyclodextrin-curcumin complexes, as a hydrophilic curcumin. They entrapped both native curcumin as a hydrophobic agent and the complexes separately into liposomes and then assessed them for their cytotoxicity in cancerous cell lines. The aqueous solubility of *β*-cyclodextrin-curcumin complexes enhanced noticeably, and successful entrapment of complexes into prepared liposomes was also achieved. The median effective dose for all curcumin formulations was found to be in a low range for both lung and colon cancer cell lines [30]. Outcomes guaranteed that *β*-cyclodextrin-curcumin complexes of weakly water-soluble drugs such as curcumin can be tricked within biocompatible vesicles such as liposomes, and this does not prevent their anticancer effects [30]. In another study, a novel curcumin analogue (difluorinated curcumin; CDF) and CDF-*β*-cyclodextrin-curcumin complex were synthesized to enhance anticancer effects against pancreatic cancer [56]. Results showed that CDF-*β*-cyclodextrin was found to lower IC50 value by half when tested against multiple cancer cell lines. Following intravenous administration of CDF-*β*-cyclodextrin, it was specially accumulated in pancreatic tissue 10 times higher than in serum. As a result, novel curcumin analogue CDF outstanding gathering in pancreas tissue led to its persuasive anticancer effects against pancreatic cancer cells. So, synthesis of such CDF-*β*-cyclodextrin self-assembly is a successful approach to improve its bioavailability and tissue distribution. Further evaluations on CDF delivery in clinical settings for treatment of human malignancies were suggested by these authors [56]. Moreover, a novel poly(*β*-cyclodextrin)-curcumin self-assembly was approached to improve curcumin's delivery to prostate cancer cells by Yallapu et al. [112]. Intracellular uptake of the self-assembly was evaluated by means of flow cytometry and immunofluorescence microscopy. The therapeutic values were established by cell proliferation and colony formation tests on prostate cancer cells. Results recommended that the poly(*β*-cyclodextrin)-curcumin formulation could be a valuable system for developing curcumin delivery and its therapeutic effectiveness in prostate cancer [112]. Additionally, in order to improve solubility and drug delivery of curcumin, Lomedasht et al. [113] exploited a *β*-cyclodextrin-curcumin inclusion complex and evaluated its cytotoxic effects by MTT assay in vitro. Breast cancer cells were treated with equal concentration of *β*-cyclodextrin-curcumin and free curcumin. Then, telomerase gene expression was compared by real-time PCR in two groups. In vitro results showed that *β*-cyclodextrin-curcumin increased curcumin delivery in breast cancer cells [113]. Telomerase gene expression was lower in *β*-cyclodextrin-curcumin-treated cells than free curcumin-treated cells. As a result, *β*-cyclodextrin-curcumin complex was more effectual than free curcumin in telomerase expression inhibition. Rocks et al. [114] have used cyclodextrins as an excipient permitting a significant enhancement of curcumin solubility and bioavailability. Then, complex's effects were evaluated in cell cultures as well as in vivo, in an orthotopic lung tumor mouse model. Cell proliferation in the presence of curcumin-cyclodextrin complex was decreased while apoptosis rates were increased in lung epithelial tumor cells in vitro. For in vivo experiments, cells were grafted into lungs of C57Bl/6 mice treated by an oral administration of a nonsoluble form of curcumin, Cds alone, or curcumin-CD complexes, combined with or not combined with gemcitabine [114]. In addition, the size of orthotopically implanted lung tumors was noticeably reduced by curcumin complex administration in comparison with nonsolubilized curcumin. Moreover, curcumin-cyclodextrin complex potentiated the gemcitabine-mediated antitumor effects. Results underlined a prospective preservative effect of curcumin with gemcitabine, thus providing a proficient remedial alternative for anti-lung cancer treatment [114]. Moreover, for noninvasive imaging, encapsulated 4-[3,5-bis(2-chlorobenzylidene-4-oxo-piperidine-1-yl)-4-oxo-2-butenoic-acid] (CLEFMA) was developed by using hydroxypropyl *β*-cyclodextrin [115]. CLEFMA possessed more persuasive antiproliferative effects in lung adenocarcinoma without any impact on normal lung fibroblasts. It seems that CLEFMA liposomes retained the antiproliferative effectiveness of free CLEFMA while sustaining its nontoxic character in normal lung fibroblasts. In addition, tumor volume extensively reduced after treatment with CLEFMA, to 94% in rat xenograft tumors. Outcomes revealed the usefulness of liposomes to supply as a carrier for CLEFMA, and this study was the first to exhibit the efficacy of novel curcuminoid CLEFMA in a preclinical model [115].

To sum up, these collected data show that Cds help increase the hydrolytic stability of curcumin, photodecomposition rate, protection against decomposition, bioavailability, and molecular dispersion compared to the free curcumin without altering their pharmacokinetic characteristics (Table 1). These data also confirm that cyclodextrin-curcumin complex has a priority against free curcumin in cell uptake, antiproliferative and anti-inflammatory effects by suppression of cyclin D1, MMP-9, and VEGF, and induction of death receptors and apoptosis.

## 7. Dendrimers

Dendrimers are a group of greatly branched globular polymers which are created with structural control rivaling traditional biomolecules. They were introduced in the mid-1980s and are referred to as synthetic proteins. Dendrimers are a series of polymeric architectures with different chemical and surface-related properties. They have much more accurately controlled structures, with a globular shape and a single molecular weight rather than a distribution of molecular weights in comparison with the traditional linear polymers [116]. A number of properties put together dendrimers' exceptional nanostructures with the interior-surface architecture or generations (Table 1). The dendrimer structure, consisting of a core, branched interiors, and numerous surface functional groups, serves as a platform to which additional substrates can be added to this spherical molecule in a highly controlled manner. This nanospace represents an isolated environment, thus decreasing toxicity associated with the payload. The well-defined organization, dense spherical form, size, monodispersity, and controllable “surface” functionalities of dendrimers make them brilliant applicants for assessment as drug delivery services [117]. In addition, the biocompatibility silhouette of dendrimers donates to their effectiveness in molecular imaging. This biocompatibility can be increased via functionalization with small molecules. Increased biocompatibility is also associated with lower generation branch cells with anionic or neutral groups compared to similar branch cells of higher generations which have cationic surface groups.

To test whether dendrimer curcumin displays both cytotoxicity and water solubility, Debnath et al. [57] generated dendrimer curcumin conjugate, a water-soluble and effective cytotoxic agent against breast cancer cell lines. In vitro results showed that dendrimer curcumin conjugate dissolved in water was significantly more effective in inducing cytotoxicity against SKBr3 and BT549 human breast cancer cells and effectively induced cellular apoptosis measured by caspase-3 activation. In another study, the interaction of curcumin dendrimers with cancer cells, serum proteins, and human red blood cells was studied by Yallapu et al. [58]. They assessed dendrimers' potential application for in vivo preclinical and clinical studies. Protein interaction studies were conducted using particle size analysis, zeta potential, and western blot techniques. To evaluate its acute toxicity and hemocompatibility, curcumin-dendrimer was incubated with human red blood cells. In addition, the cellular uptake of curcumin-dendrimer was assessed by using curcumin levels in cancer cells using ultraviolet-visible spectrophotometry. Results showed a remarkable capacity of the dendrimer curcumin nanoformulation to bind to plasma protein. However, no significant changes were observed in the zeta potential and the extensive hemolysis of the dendrimer curcumin formulation. Results showed that the positively charged amino surface groups cause destabilize the cell membrane and cell lysis. This type of lytic effect on erythrocytosis is extremely dangerous when administered in vivo. Therefore, polyethylene glycol conjugation of dendrimer formulations may be required to decrease this activity [118, 119].

Cao et al. [59] investigated the interactions between polyamidoamine-C (a dendrimers) and curcumin by using fluorescence spectroscopy and molecular modeling methods. Results showed that the polyamidoamine-C12 25% formation together with curcumin induced the fluorescence quenching of polyamidoamine-C12 25%. Curcumin entered the interface of polyamidoamine-C12 25% with mainly five classes of binding sites by hydrophobic bonds, hydrogen bonds, and van der Waals forces interactions. The larger values of binding constants indicated that polyamidoamine-C12 25% holds the curcumin strongly. Furthermore, in another study, polyamidoamine encapsulated curcumin inhibited telomerase activity in human breast cancer cell line [60]. These researchers also used telomerase repeat amplification protocol (TRAP) assay and determined relative telomerase activity (%RTA). In vitro results demonstrated that dendrimers have no cytotoxicity in human breast cancer cell line. Also, polyamidoamine encapsulating curcumin concentration increased while %RTA decreased. These results suggested that polyamidoamine encapsulating curcumin had a dose-dependent cytotoxicity effect on breast cancer cell line through downregulation and inactivation of telomerase and inducing apoptosis by enhancing curcumin uptake by cells (Table 1). So, polyamidoamine can be considered as a fine carrier especially for hydrophobic agents.

The stability of curcumin and its antitumor properties were improved by using dendrosomal nanoparticles in vitro and in vivo by our team's work [61–63, 120]. The made dendrosomal nanoparticle-curcumin is a neutral, amphipathic, and biodegradable nanomaterial with variable monomers suitable for inert cell drug porters. It is a new type of biocompatible polymeric particle taken from plant fatty acids which keeps curcumin size at 80 nm (Table 1). Acute and chronic toxicity of dendrosomal nanoparticle-curcumin was investigated in mice. Our results shed new light on dendrosomal nanoparticle-curcumin's potential biocompatibility for in vitro and in vivo biological systems. In addition, the protective and the therapeutic effects of dendrosomal nanoparticle-curcumin were assessed on an animal model of breast cancer through apoptosis, proliferation, and angiogenesis pathways. In our study, dendrosomal nanoparticle-curcumin significantly suppressed proliferation of human and mouse carcinoma cells. In vitro results showed not only that dendrosomes have significantly increased the uptake of curcumin but also that dendrosomal nanoparticle-curcumin inhibited the growth of cancer cells rather than normal ones by inducing apoptosis. In toxicity profile, based on hematological, blood chemical, and histological examinations, minimal hepatic and renal toxicity were seen with high dendrosomal nanoparticle-curcumin doses. In addition, in vivo results showed that tumor incidence, weight, and size were significantly declined in dendrosomal nanoparticle-curcumin-treated group. Dendrosomal nanoparticle-curcumin also induced the expression of proapoptotic Bax protein and reduced antiapoptotic Bcl-2 protein expression relative to the control group. Moreover, proliferative and angiogenic markers were lowered in dendrosomal nanoparticle-curcumin-treated animals. These findings point to the features of the polymeric carrier as a promising drug-delivery system for cancer therapy. In another study, we also evaluated the antiproliferative and anticarcinogenic effects of dendrosomal nanoparticle-curcumin in rat colon cancer. Our results demonstrated the potential anticancer effects of dendrosomal nanoparticle-curcumin in a typical animal model of colon cancer. The results provide evidence that nanoparticle-curcumin exerts significant chemoprotective and chemotherapeutic effects on colon cancer through inhibition of cell proliferation and apoptosis induction [61, 63]. These tunable properties make dendrimers more attractive agents for biomedical applications compared to other nanovectors such as micelles, liposomes, or emulsion droplets (Table 1). Therefore, they are being preferred as carriers which are the foundation for new types of anticancer entities. Although the application of dendrimers as drug-delivery instruments has been advertised as a major area of their potential application, this part has really been little studied [121].

So, mentioned studies suggest that dendrimer curcumin conjugate in water was significantly more effective in inducing cytotoxicity through downregulation and inactivation of telomerase activity and in inducing apoptosis by induction of the expression of proapoptotic Bax protein and reduction of antiapoptotic Bcl-2 protein expression since curcumin uptake enhances.

## 8. Nanogels

Nanogels are self-possessed of cross-linked three-dimensional polymer chain networks which are created through covalent linkages and can be customized to gel networks with biocompatible and degradable properties. The porosity among these cross-linked networks not only provides a perfect reservoir for loading drugs but also keeps them from environmental degradation [58]. The swelling of nanogels in an aqueous setting is controlled by using the polymer chemical structure, cross-linking degree, and the polyelectrolyte gel's charge density and/or by pH value, ionic strength, and chemical nature of low molecular mass (Table 1). Furthermore, nanogels can be chemically modified to incorporate various ligands for targeted drug delivery, triggered drug release, or preparation of composite materials [122].

Nanogels are developed as carriers for drug delivery and can be planned to spontaneously absorb biologically active molecules via creation of salt bonds, hydrogen bonds, or hydrophobic interactions that can enhance oral and brain bioavailability of low-molecular-weight drugs and biomacromolecules [122]. An important criterion for a nanogel carrier with widespread biomedical abilities is to have good stability in biological fluids, which would prohibit aggregation. In this regard, Gonçalves et al. (2012) applied a self-assembled dextrin nanogel as curcumin delivery system by using dynamic light scattering and fluorescence measurements. They showed that the stability and loading efficiency of curcumin-loaded nanogel depend on the nanogel/curcumin ratio. The in vitro release profile in HeLa cell cultures indicated that dextrin nanogel may act as a suitable carrier for the controlled release of curcumin [123]. Various nanogel properties can be attained by altering the chemical functional groups, cross-linking density, and surface-active and stimuli-responsive elements [58]. Nanogels demonstrate excellent potential for systemic drug delivery that should have a few common features including a smaller particle size (10–200 nm), biodegradability and/or biocompatibility, prolonged half-life, high stability, higher amount of drug loading and/or entrapment, and molecules protection from immune system [58]. Mangalathillam et al. (2011) loaded curcumin into chitin nanogels and analyzed it by dynamic light scattering (DLS), scanning electron microscope (SEM), and Fourier transform infrared spectroscopy (FTIR). Then, the nanogel's cytotoxicity was analyzed on human dermal fibroblast and human melanoma cells. The curcumin-chitin nanogels showed higher release at acidic pH compared to neutral pH. The in vitro results showed that curcumin-chitin nanogels have had a specific toxicity on melanoma cells in a concentration range of 0.1–1.0 mg/mL, but less toxicity towards normal cells [64]. The confocal analysis confirmed the high uptake of curcumin-chitin nanogels by human melanoma cells. In addition, it was indicated that curcumin-chitin nanogels at the higher concentration of the cytotoxic range may show comparable apoptosis in comparison with free curcumin. The curcumin-chitin nanogels also showed a 4-fold increase in steady state transdermal flux of curcumin in comparison with free curcumin. The histopathology studies showed loosening of the horny layer of the epidermis, facilitating penetration with no observed signs of inflammation in the group treated with curcumin-chitin nanogels [64]. These results suggested the formulated curcumin-chitin nanogels' explicit advantage for the treatment of melanoma by effective transdermal penetration.

Drug release from nanogels' networks depends on the interaction of hydrophobic and hydrogen complication and/or coordination of drug molecules with the polymer chain networks. Preclinical studies suggest that nanogels can be used for the efficient delivery of biopharmaceuticals in cells as well as for increasing drug delivery across cellular barriers [124]. Wu et al. [125] designed a class of water-dispersible hybrid nanogels for intracellular delivery of hydrophobic curcumin. They synthesized hybrid nanogels by coating the Ag/Au bimetallic nanoparticles with a hydrophobic polystyrene gel layer as internal shell and a subsequent thin hydrophilic nonlinear poly(ethylene glycol-) based gel layer as external shell. The Ag/Au core nanoparticles not only emitted well-built fluorescence for imaging and monitoring at the cellular level but also exhibited burly absorption in the near-infrared region for photothermal conversion and significantly improved the therapeutic efficacy. Furthermore, while the internal polystyrene gel layer was introduced to provide strong hydrophobic interactions with curcumin for high drug loading yields, the external nontoxic and thermoresponsive poly(ethylene glycol) analog gel layer was designed to trigger the release of the preloaded curcumin by either variation of surrounding temperature or exogenous irradiation with near-infrared light. These results suggest that such designed multifunctional hybrid nanogels are properly suited for in vivo and clinical trials by promising natural medicine of curcumin to the forefront of therapeutic agents for cancers and other diseases. In addition, hyaluronic acid- (HA-) based nanogel-drug conjugates with enhanced anticancer activity were designed by Wei et al. for the targeting of CD44-positive and drug-resistant tumors [65]. These authors synthesized nanogel-drug conjugates based on membranotropic cholesteryl-HA for efficient targeting and suppression of drug-resistant tumors. This class of tumors expresses CD44 receptors, cellular glycoproteins which bind to HA. These nanogel conjugates have significantly increased the bioavailability of poorly soluble drugs such as curcumin. In this study, the small nanogel particles with a hydrophobic core and high drug loads were formed after ultrasonication [65]. These nanogel particles demonstrated a sustained drug release following the hydrolysis of biodegradable ester linkage. Importantly, cholesteryl-HA-drug nanogels demonstrated a 2–7 times higher cytotoxicity in CD44-expressing drug-resistant human breast and pancreatic adenocarcinoma cells [65]. These nanogels were efficiently internalized via CD44 receptor-mediated endocytosis and simultaneous interaction with the cancer cell membrane [65]. Anchoring by cholesterol moieties in cellular membrane caused more efficient drug accumulation in cancer cells. The cholesteryl-HA nanogels were able to penetrate multicellular cancer spheroids and exhibited a higher cytotoxic effect in the system modeling tumor environment than both HA-drug conjugates and free drugs [65].

Overall, the proposed design of nanogel-drug conjugates can allow significantly enhancing drug bioavailability, stability, loading efficiency, effective transdermal penetration, cancer cell targeting, and treatment efficacy against drug-resistant cancer cells and multicellular spheroids (Table 1).

## 9. Chitosans

Chitosan is a linear polysaccharide composed of randomly disseminated deacetylated and acetylated units. It is made commercially by deacetylation of chitin, which is the structural component of crustaceans' exoskeleton and fungi cell walls. Unlike other biodegradable polymers, chitosan is the only one exhibiting a cationic character due to its primary amino groups that responsible for various effects in drug delivery systems [126]. It displays particular properties, for example, solubility in various media, polyoxysalt creation, polyelectrolyte behavior, metal chelations, and structural uniqueness (Table 1). One study showed that the fluorescence intensity of curcumin can be greatly improved in the presence of chitosan by bovine and human serum albumin [104]. The method has been profitably used for the determination of human serum albumin in real samples. Data analysis recommended that the highly enhanced fluorescence of curcumin resulted from synergic effects of favorable hydrophobic microenvironment provided by bovine serum albumin and chitosan and efficient intermolecular energy transfer between bovine serum albumin and curcumin. Bovine serum albumin may bind to chitosan through hydrogen bonds, which causes the protein conformation to switch from *β*-fold to *α*-helix. Curcumin can combine with bovine serum albumin from *β*-fold to *α*-helix and can also combine with the bovine serum albumin-chitosan complex via its center carbonyl carbon. Therefore, chitosan plays a key role in promoting the energy transfer process by shortening the distance between bovine serum albumin and curcumin [104].

Polycaprolactone nanocarriers decorated with a mucoadhesive polysaccharide chitosan containing curcumin were also developed [127]. In order to optimize the preparation conditions, these nanocarriers were prepared by the nanoprecipitation method by using different molar masses and concentrations of chitosan and triblock surfactant poloxamer. Chitosan-coated nanocarriers revealed positive surface charge and a mean particle radius ranging between 114 and 125 nm, confirming the decoration of the nanocarriers with the mucoadhesive polymer, through hydrogen bonds between ether and amino groups, from poloxamer and chitosan, respectively. Dynamic light scattering studies have shown monodisperse nanocarriers. Furthermore, colloidal systems showed mean drug content about 460 lg/mL and encapsulation efficiency higher than 99%. In summary, these nanocarriers showed a vast ability to interact with mucin, also indicating their suitability for mucoadhesive applications when coated with chitosan [127].

On the other hand, curcumin-phytosome-loaded chitosan microspheres were developed by combining polymer- and lipid-based delivery systems to improve the bioavailability and prolong the retention time of curcumin [66]. These complexes were produced by encapsulating curcumin phytosomes in chitosan microspheres using ionotropic gelation. Differential scanning calorimetry and FUTI spectroscopy revealed that the integrity of the phytosomes was protected within the polymeric matrix of the microspheres. In vitro release rate of curcumin from the curcumin-phytosome-loaded chitosan microspheres was slower than curcumin-loaded chitosan microspheres. Pharmacokinetic studies showed an increase in curcumin absorption in curcumin-phytosome-loaded chitosan microspheres compared with curcumin phytosomes and curcumin-loaded chitosan microspheres. Moreover, half-life of curcumin in oral administration of curcumin-phytosome-loaded chitosan microspheres was longer than the two other ones. These results indicated that the novel curcumin-phytosome-loaded chitosan microspheres combined system has the advantages of both the chitosan microspheres and the phytosomes, which had better effects of promoting oral absorption and prolonging retention time of curcumin than single curcumin phytosomes or curcumin-loaded chitosan microspheres. Therefore, the phytosome chitosan microspheres may be used as a sustained delivery system for lipophilic compounds with poor water solubility and low oral bioavailability [66]. A study showed that curcumin bound to chitosan nanoparticles was not rapidly degraded in comparison to free curcumin, and the uptake of curcumin-loaded chitosan NPs by mouse's red blood cells (RBC) was much better than free curcumin [67]. Oral delivery of curcumin-loaded chitosan NPs improved the bioavailability of curcumin both in plasma and in RBC. Like chloroquine, conjugated curcumin inhibited parasite lysate induced heme polymerization in vitro in a dose dependent manner, and it had a lower IC50 value than chloroquine. Additionally, feeding of curcumin-loaded chitosan NPs caused a higher survival in mice infected with a lethal strain of* Plasmodium yoelii*. Therefore, binding of curcumin to chitosan NPs improves its chemical stability and bioavailability. In vitro data also suggest that this complex can inhibit hemozoin synthesis which is lethal for the parasite [67].

In another study, chitosan showed promising features as auxiliary agent in drug delivery (e.g., slimming, wound dressing, and tissue engineering). An in situ injectable nanocomposite hydrogel curcumin was effectively developed for use as a treatment in the dermal wound repair process [68]. In vitro release studies disclosed that the encapsulated nanocurcumin was slowly released from the N,O-carboxymethyl chitosan/oxidized alginate hydrogel with the controllable diffusion behavior. Additionally, in vivo wound healing studies revealed that application of nanocurcumin/N,O-carboxymethyl chitosan/oxidized alginate hydrogel could significantly improve the reepithelialization of epidermis and collagen deposition on rat dorsal wounds. DNA, protein, and hydroxyproline content in wound tissue indicated that making a combination by using nanocurcumin and N,O-carboxymethyl chitosan/oxidized alginate hydrogel could significantly accelerate the process of wound healing. So, results suggested that the developed nanocurcumin/N,O-carboxymethyl chitosan/oxidized alginate hydrogel as a promising wound dressing might have potential application in the wound healing [68].

Water-soluble nanocarriers of curcumin were synthesized, characterized, and applied as a stable detoxifying agent for arsenic poisoning [69]. The therapeutic efficacy of encapsulated curcumin nanocarriers was investigated against arsenic-induced toxicity in an animal model. In this regard, sodium arsenite and encapsulated curcumin were orally administered to male Wistar rats for 4 weeks. Arsenic dramatically declined blood d-aminolevulinic acid dehydratase activity and glutathione and increased blood reactive oxygen species. These alterations were accompanied by increases in hepatic total ROS, oxidized glutathione, and thiobarbituric acid-reactive substance levels. By contrast, hepatic glutathione, superoxide dismutase, and catalase activities were considerably declined after arsenic exposure, indicative of oxidative stress. Brain amines levels such as dopamine, norepinephrine, and 5-hydroxytryptamine also showed considerable changes after arsenic exposure. Coadministration of encapsulated curcumin nanocarriers provided obvious favorable effects on the adverse changes in oxidative stress parameters induced by arsenic. The results revealed that encapsulated curcumin nanocarriers have better antioxidant and chelating potential compared to free curcumin. Therefore, the significant neurochemical and immunohistochemical protection afforded by encapsulated curcumin nanocarriers shows their neuroprotective effectiveness [69]. Chitosan also explains fungistatic, haemostatic, and antitumor effects [70]. In this regard stable vesicles for efficient curcumin encapsulation, delivery, and controlled release have been obtained by coating of liposomes with thin layer of newly synthesized chitosan derivatives [71]. Some special derivatives of chitosan were studied such as the cationic, hydrophobic, and cationic-hydrophobic derivatives. Zeta potential data proved effectual coating of liposomes with all these derivatives. In this regard, the liposomes coated with cationic-hydrophobic chitosan derivatives were the main promising curcumin carriers. They can easily enter cell membrane and release curcumin in a controlled approach, and the biological investigations showed that such organizations are nontoxic for normal murine fibroblasts while toxic for murine melanoma tumors [71].

In a recent study, Pluronic F127 was used to enhance the solubility of curcumin in the alginate-chitosan NPs [128]. Atomic force and scanning electron microscopic analysis demonstrated that the particles were almost spherical in shape (100 ± 20 nm). Fourier transform infrared analysis showed impending interactions among the components in the composite NPs. Furthermore, encapsulated curcumin efficiency confirmed considerable increase over alginate-chitosan NPs without Pluronic. Cytotoxicity assay explained that composite NPs at a concentration of 500 *μ*g/mL were nontoxic for HeLa cells. Moreover, cellular internalization of curcumin-loaded complex was confirmed by green fluorescence inside the HeLa cells [128]. Curcumin-loaded biodegradable thermoresponsive chitosan-g-poly copolymeric NPs were prepared by using ionic cross-linking method [129]. The results showed that these NPs were nontoxic to different cancerous cell lines, whereas the curcumin loaded with NPs showed a specific toxicity for the abovementioned cell lines. Additionally, these results were further approved by flow cytometry analysis which proved increased apoptosis on these cell lines in a concentration-dependent manner. Furthermore, the blood compatibility assay showed the possibility of an IV injection with this formulation. Preliminary study provided clear evidence for the thermal targeting of curcumin by being loaded with novel thermosensitive chitosan-g-PNIPAAm NPs, and efficacies were achieved in cancer therapy. These results indicated that thermoresponsive chitosan-g-poly copolymeric NPs can be a potential nanocarrier for curcumin drug delivery [129]. Novel cationic poly(butyl) cyanoacrylate (PBCA) NPs coated with chitosan were synthesized with curcumin. The transmission electron microscopy showed the spherical shape of prepared NPs along with the particle size. Curcumin NPs demonstrated more therapeutic efficacy than free curcumin against a panel of human hepatocellular cancer cell lines. Encapsulated curcumin with PBCA NPs caused a profound change in the pharmacokinetics of the drug. The elimination half-life of curcumin was increased 52-fold in loaded form with PBCA NPs, and ultimately its clearance was also decreased 2.5-fold. Additionally, the higher plasma concentration of curcumin for curcumin-PBCA NPs might be a result of the NPs size and chitosan coating to keep drug in the blood circulation for a more extended period. Besides, the mean residence time of curcumin-PBCA NPs was longer than free curcumin. These results might be due to accumulation of NPs in endoplasmic reticulum system of organs and sustained release of the drug from them. Furthermore, the carriers' properties, for instance, shape, size, charge, and hydrophilicity, can prolong the retention of them in the blood circulation. There was also a substantial increase in the distribution volume (51-fold) that was quite unexpected. Obviously, it was possible that the larger micellar carriers were sequestered by the reticuloendothelial system or other tissues and truly led to improved distribution volume [130]. Additionally, treatment with curcumin NPs resulted in reduced tumor size and visible blanching of tumors [131].

So far, curcumin-loaded chitosan NPs improve the bioavailability and prolong the retention time of curcumin due to accumulation of NPs in endoplasmic reticulum system and the carriers' features such as shape, size, charge, and hydrophilicity (Table 1). Gathered data also propose that this complex can be lethal for the parasite because of hemozoin synthesis inhibition. Some in vivo experiments also resulted in better wound healing after application of curcumin-loaded chitosan NP polymers by means of better reepithelialization of epidermis and collagen deposition. This complex could also be administered in order to detoxify arsenic through better antioxidant and chelating potential. These compounds gained some achievements in cancer therapy as well.

## 10. Gold Nanoparticles

Metal nanoparticles have been known since very old times, and gold nanoparticles (AuNPs) with optical and electrochemical uniqueness have proven to be a potent apparatus in nanomedicinal requests [132]. They have also been largely used in immunochemistry, immunohistochemistry, and immunoblotting for electron microscopy. They are often generated in various shapes [132], and their properties are strongly dependent on the conditions in which they are prepared. Moreover, the stability of AuNPs and their capability to combine with biomolecules are their other outstanding properties. AuNPs are studied broadly as imperative drug delivery vectors due to some of their characteristic aspects, such as low cytotoxicity, tunable surface features, and stability in in vivo conditions, and can be easily synthesized and functionalized (Table 1). They can also act as drug pool for small drug molecules, proteins, DNA, or RNA with improved long life in the blood circulation. Rajesh et al. [133] used polyvinyl pyrrolidone (PVP) as a proven drug carrier to curcumin conjugation with AuNPs to enhance solubility of curcumin. Results showed a superior assurance for such conjugates as therapeutic-curcumin-imaging materials in biomedical field [134]. Kumar et al. (2012) also prepared the chitosan-curcumin nanocapsules with AuNPs via solvent evaporation method. Scanning electron microscopy and transmission electron microscopy were done to describe the drug entrapped nanocapsules. The average diameter of AuNPs was found to be in the range of 18–20 nm, and the nanocapsules were found to be in the range of 200–250 nm. Furthermore, the Fourier transform infrared analysis revealed no possible interactions among the constituents with the chitosan nanoparticles. The drug release studies revealed that curcumin encapsulated chitosan with AuNPs was controlled and steadied when compared with curcumin encapsulated chitosan nanoparticles. Use of in vitro drug release in various kinetic equations indicated a matrix model with uniform distribution of curcumin in the nanocapsules [135]. Additionally, the tunability of AuNPs allows for complete control of surface properties for targeting and sustained release of the bioactive molecules [136].

In a study by Singh et al. [72] curcumin was bound on the surface of AuNPs in order to increase the bioavailability of it. The AuNPs were synthesized by direct decline of HAuCl4 by curcumin in aqueous part. Curcumin acted as both a reducing and capping agent and a stabilizing gold sol for many months. Furthermore, these curcumin-capped AuNPs showed an excellent antioxidant activity which was established by 2,2-diphenyl-l-picrylhydrazyl radical test. Consequently, the practical surface of AuNPs with curcumin may suggest a new way of use of curcumin towards possible drug delivery and therapeutics [72]. In another study, effect of curcumin-conjugated-AuNPs was investigated on peripheral blood lymphocytes [137]. The treated lymphocytes showed typical characteristics of apoptosis which included chromatin condensation and membrane blebbing and occurrence of apoptotic bodies. Results revealed that these conjugated nanoparticles may be used as drugs in nontoxic range [137]. In order to target cancer at a single cell level, gold-citrate nanoparticles were also synthesized with diameters of 13 nm [73]. AuNPs were coated with sodium citrate. Outcomes revealed that cancerous cells were more prone to absorb nanomaterials coated with citrate than normal somatic cells. Moreover, the damage was reversible with AuNPs and the normal dermal fibroblast cells were able to regenerate stress fibers which were lost during exposure. However, cancer cells were unable to recover from the damage inflicted by Au/citrate nanoparticle exposure [73]. Manju and Sreenivasan [136] also formulated a simple method for the fabrication of water-soluble curcumin conjugated AuNPs to target various cancer cell lines. Curcumin conjugated to hyaluronic acid to get a water-soluble compound. They were made AuNPs by diminishing chloroauric acid using hyaluronic acid-curcumin, which played dual roles of a reducing and a stabilizing agent and subsequently anchored folate conjugated PEG. Their interaction with various cancer cell lines was followed by flow cytometry and confocal microscopy. Blood-materials interactions studies proved that the nanoparticles are extremely hemocompatible. Flow cytometry and confocal microscopy results demonstrated considerable cellular uptake and internalization of the particles by various cancer cells [136].

In conclusion, curcumin conjugated AuNPs exhibited more cytotoxicity compared to free curcumin (Table 1). AuNPs also cause targeting and sustained release of curcumin and an excellent antioxidant activity.

## 11. Silvers

Silver has usually been utilized as an incredibly efficient material for antimicrobial utility [138]. In small concentrations, it is safe for human cells but lethal for the majority of bacteria and viruses [139]. With development of nanotechnology, it has become the metal of choice in restricting microbial growth and expansion in a variety of nanoparticle-related requests [138]. Silver nanoparticles are identified for their brilliant optoelectronic properties originated from surface plasmon resonance. They can be used in optoelectronics, biological labeling, and biological and chemical sensing (Table 1). They have shown excellent antimicrobial activity compared to other available silver antimicrobial agents.

Sodium carboxylmethyl cellulose silver nanocomposite films were attempted for antibacterial applications, so, to improve their applicability, novel film-silver nanoparticle-curcumin complexes have been developed [74]. These films were described by FTIR, UV-visible, X-ray diffraction (XRD), thermogravimetric analysis (TGA), differential scanning calorimetry (DSC), and TEM techniques. The structured silver nanoparticles had a typical particle size of 15 nm. Curcumin loading into sodium carboxylmethyl cellulose silver nanocomposite films was achieved by diffusion mechanism. The UV analysis showed superior encapsulation of curcumin in the films with higher sodium carboxylmethyl cellulose content. Additionally, it was surveyed that the presence of silver nanoparticles in the films improved the encapsulation of curcumin demonstrating an interaction between them. Moreover, results showed that the sodium carboxylmethyl cellulose films produced with silver nanoparticles have a synergistic effect in the antimicrobial activity against* E. coli*. Furthermore, curcumin loaded with sodium carboxylmethyl cellulose silver nanocomposite films extended considerable inhibition of* E. coli* growth compared with the silver nanoparticles and curcumin alone film. Therefore, the study obviously supplied novel antimicrobial films which were potentially helpful in preventing/treating infections [74]. In another study, novel hydrogel-silver nanoparticle-curcumin composites have been built up to increase its applicability. These were first synthesized by polymerizing acrylamide in the presence of polyvinyl sulfonic acid sodium salt and a trifunctional cross-linker (2,4,6-triallyloxy 1,3,5-triazine) by using redox initiating system. Silver nanoparticles were then produced throughout the hydrogel networks by using in situ method incorporating the silver ions and following drop with sodium borohydride. Curcumin loading into hydrogel-silver nanoparticles complex was earned by diffusion mechanism. An attractive arrangement of silver nanoparticles (shining sun ball in range 5 nm) with apparent smaller grown nanoparticles (1 nm) was detected. A comparative antimicrobial study was performed for hydrogel-silver nanocomposites and hydrogel-silver nanoparticle-curcumin composites. The results indicated that hydrogel-Ag NPs-curcumin composites have exhibited greater reduction of* E. coli* growth compared with Ag NPs loaded hydrogels. The current work demonstrated that combining hydrogel, nanotechnology, and curcumin is promising for developing novel antimicrobial agents with potential applications in dressing of various types of skin wounds. The entrapped silver nanoparticles and curcumin molecules showed sustained release which advises enormous prolonged therapeutic values [74]. In addition, silver nanoparticles could protect cells against HIV-1 infection and help with the wound healing process and also have essential function as an anti-inflammation, an antiviral, and an anticancer agent [75]. So, the combination of silver nanoparticles and curcumin, besides prolonged therapeutic outcomes and sustained release, has several other useful effects such as anti-inflammatory, anti-infection, anticancer, and wound healing (Table 1).

## 12. Solid Lipids

Solid lipid nanoparticles (SLNs) are one of the novel potential colloidal carrier systems as alternative materials to polymers for parenteral nutrition. SLNs have typically spherical and submicron colloidal carriers (50 to 1000 nm) and are composed of physiologically tolerated lipid components with solid shape at room temperature (Table 1). They are one of the most fashionable advances to develop the oral bioavailability of poorly water-soluble drugs [76]. Advantages of SLNs are high and improved drug content, ease of scaling up and sterilizing, better control over release kinetics of encapsulated compounds, enhanced bioavailability of entrapped bioactive compounds, chemical protection of incorporated compounds, much easier manufacturing than biopolymeric nanoparticles, conventional emulsion manufacturing methods, and applicability and very high long-term stability application versatility [76].

Kakkar et al. [77] loaded curcumin into SLNs to improve its oral bioavailability. Curcumin-SLNs with an average particle size of 134.6 nm and a total drug content of <92% were produced by using a microemulsification technique. In vivo pharmacokinetics was performed after oral administration of curcumin-SLNs by using a validated LC-MS/MS method in rat's plasma. Results revealed significant improvement in bioavailability times after administration of curcumin-SLNs with respect to curcumin-solid lipid. Data confirmed that enhanced and reliable bioavailability will help in establishing its therapeutic impacts [77]. Furthermore, Kakkar et al. [78] incorporated curcumin into SLNs to achieve a significant bioavailability of curcumin. Then, the plasma and brain cryosections were observed for fluorescence under fluorescent/confocal microscope. Biodistribution study was also performed using 99m Tc-labeled curcumin-SLNs and curcumin-solid lipid in mice after oral and intravenous administration. Presence of yellow fluorescent particles in plasma and brain indicated effective delivery of curcumin-SLNs across the gut wall and the blood brain barrier. Blood AU coral value for curcumin-SLNs was 8.135 times greater than curcumin-solid lipid, confirming a prolonged circulation of the former. The ratio of blood AUC intravenous curcumin-SLN/curcumin-solid lipid in blood was ≤1 while the ratio in brain promisingly indicates 30 times higher preferential distribution of curcumin-SLNs into brain confirming their direct delivery [78].

Dadhaniya et al. (2011) examined the adverse effects of a new solid lipid curcumin particle in rats. Administration of the conjugated curcumin showed no toxicologically significant treatment-related changes in the clinical parameters including behavioral observations, ophthalmic examinations, body weights and weight gains, food consumption, and organ weights or the paraclinical parameters including hematology, serum chemistry, and urinalysis. In addition, terminal necropsy revealed no treatment-related gross or histopathology findings [140]. Expansion of SLNs is one of the promising fields of lipid nanotechnology with several potential applications in drug delivery system and clinical medicine and research. The experimental paradigm of cerebral ischemia in rats by curcumin-SLNs was prepared; there was an improvement of 90% in cognition and 52% inhibition of acetylcholinesterase versus cerebral ischemic and neurological scoring, which improved by 79% [78]. Levels of superoxide dismutase, catalase, glutathione, and mitochondrial complex enzyme activities were also significantly increased, while lipid peroxidation, nitrite, and acetylcholinesterase levels decreased after curcumin-SLNs administration. Gamma-scintigraphic studies showed 16.4 and 30 times improvement in brain bioavailability upon oral and intravenous administration of curcumin-SLNs versus curcumin-silver. Results indicated the protective role of curcumin-SLNs against cerebral ischemic insult suggesting that it is packaged suitably for improved brain delivery [78]. Moreover, simultaneous curcumin treatment during the induction of neurotoxicity by aluminum was reported by Kakkar and Kaur (2011). They prepared solid lipid nanoparticles of curcumin with enhanced bioavailability and examined its therapeutic effects in alleviating behavioral, biochemical, and histochemical changes in mice. Adverse effects of aluminum were completely reversed by oral administration of curcumin-SLNs. Treatment with free curcumin showed <15% recovery in membrane lipids and 22% recovery in acetylcholinesterase with respect to aluminum treated group. Histopathology of the brain sections of curcumin-SLNs treated groups also indicated significant improvement [141]. This study emphasized the potential of curcumin-SLNs for treatment of Alzheimer's disease; though, the therapeutic potential of curcumin in terms of reversing the neuronal damage, once induced, is limited due to its compromised bioavailability [141].

Yadav et al. (2009) also developed a novel formulation approach for treating experimental colitis in the rat model by a colon-specific delivery approach. Solid lipid microparticles of curcumin were prepared with palmitic acid, stearic acid, and soya lecithin, with an optimized percentage of poloxamer 188. Then, the colonic delivery system of solid lipid microparticles formulations of curcumin was further investigated for their antiangiogenic and anti-inflammatory activities by using chick embryo and rat colitis models. Data showed that solid lipid microparticles of curcumin proved to be a potent angioinhibitory compound in the chorioallantoic membrane assay. Rats treated with curcumin and its solid lipid microparticle complex showed a faster weight gain compared with dextran sulfate solution control rats. The increase in whole colon length appeared to be significantly greater in solid lipid microparticle-treated rats when compared with free curcumin and control rats. Moreover, decreased mast cell numbers was observed in the colon mucosa of curcumin-solid lipid microparticle treated rats. The degree of colitis caused by administration of dextran sulfate solution was significantly attenuated by colonic delivery of curcumin-solid lipid microparticles [79]. Being a nontoxic natural dietary product, it seems that curcumin can be useful in the therapeutic strategy for inflammatory bowel disease patients. Wang et al. (2012) aimed to formulate curcumin-SLNs to improve its therapeutic efficacy in an ovalbumin-induced allergic rat model of asthma. in vitro tests were performed in order to check Physiochemical properties of curcumin-SLNs and its release experiments. The pharmacokinetics in tissue distribution and the therapeutic effects were studied in mice. X-ray diffraction analysis revealed the amorphous nature of the encapsulated curcumin. The curcumin concentrations in plasma suspension were considerably superior to free curcumin, and all the tissue concentrations of curcumin increased after curcumin-SLNs administration, especially in lung and liver. In addition, curcumin-SLNs efficiently suppressed airway hyperresponsiveness and inflammatory cell infiltration. It also inhibited the expression of T-helper-2-type cytokinesin bronchoalveolar lavage fluid significantly compared to free curcumin. These observations imply that curcumin-SLNs can be a promising candidate for asthma therapy [80]. In another study, transferrin-mediated SLNs were prepared to increase photostability and anticancer activity of curcumin against breast cancer cells in vitro [81]. Microplate analysis and flow cytometry techniques were used for cytotoxicity and apoptosis studies. The physical characterization showed the suitability of preparation method. Transmission electron microscopy and X-ray diffraction studies revealed the spherical nature and entrapment of curcumin in amorphous form, respectively. Annexin V-FITC/PI double staining, DNA analysis, and reduced mitochondrial potential confirmed the occurrence of apoptosis. The flow cytometric studies disclosed that the anticancer activity of curcumin is enhanced with transferrin-mediated SLNs compared to free curcumin, and apoptosis is the mechanism underlying the cytotoxicity (Table 1). Results indicated the potential of transferrin-mediated SLNs in enhancing the anticancer effect of curcumin in breast cancer cells in vitro [81].

## 13. Conclusion and Future Perspectives

The use of nanotechnology in medicine and more purposely drug delivery is set to spread quickly. Currently, many substances are under investigation for drug delivery and more specifically for cancer therapy. Fascinatingly, pharmaceutical sciences are using nanoparticles to reduce toxicity and side effects of drugs. Moreover nanoparticles augment solubility and stability of some substances like curcumin. It is now clear that further development of traditional natural compounds with chemopreventive and chemotherapeutic potential such as curcumin will be dictated by the advanced drug delivery systems. Nanotechnology is assumed to be a fundamental setting in drug delivery system and human therapeutics. However, considerable challenges remain in driving this field into clinically practical therapies. Curcumin, an excellent representative derived from traditional natural compounds, has been proven to be effectual in long-term application and preclinical trials. There is no doubt that advance of novel delivery systems of curcumin with better therapeutic effects will be vital for future improvement of curcumin as a therapeutic agent. Thus, it is an enormous implication to overcome the current limitations of curcumin. It seems that only by multidisciplinary collaboration we can bring these promising traditional natural compounds to the forefront of therapeutic agents for different diseases. Therefore, the promise of nanotechnology-based medicine may become a reality with sufficient efforts and further researches. Human trials need to be conducted to establish curcumin's effectiveness in clinical applications as an improved therapeutic modality for treatment of different diseases.

## Figures and Tables

**Table 1:** Nanoparticles-conjugated curcumin characterization for different diseases treatment.